# Direct impact of cisplatin on mitochondria induces ROS production that dictates cell fate of ovarian cancer cells

**DOI:** 10.1038/s41419-019-2081-4

**Published:** 2019-11-07

**Authors:** Markus Kleih, Kathrin Böpple, Meng Dong, Andrea Gaißler, Simon Heine, Monilola A. Olayioye, Walter E. Aulitzky, Frank Essmann

**Affiliations:** 1 Dr. Margarete-Fischer-Bosch Institute of Clinical Pharmacology and University of Tuebingen, Stuttgart, Germany; 2 Institute of Cell Biology and Immunology, University of Stuttgart, Stuttgart, Germany; 3 Department of Hematology and Oncology, Robert-Bosch-Hospital, Stuttgart, Germany

**Keywords:** Cancer, Cancer, Cancer metabolism, Cancer metabolism, Preclinical research

## Abstract

Patients with high-grade serous ovarian cancer (HGSC) frequently receive platinum-based chemotherapeutics, such as cisplatin. Cisplatin binds to DNA and induces DNA-damage culminating in mitochondria-mediated apoptosis. Interestingly, mitochondrial DNA is critically affected by cisplatin but its relevance in cell death induction is scarcely investigated. We find that cisplatin sensitive HGSC cell lines contain higher mitochondrial content and higher levels of mitochondrial ROS (mtROS) than cells resistant to cisplatin induced cell death. In clonal sub-lines from OVCAR-3 mitochondrial content and basal oxygen consumption rate correlate with sensitivity to cisplatin induced apoptosis. Mitochondria are in two ways pivotal for cisplatin sensitivity because not only knock-down of BAX and BAK but also the ROS scavenger glutathione diminish cisplatin induced apoptosis. Mitochondrial ROS correlates with mitochondrial content and reduction of mitochondrial biogenesis by knock-down of transcription factors PGC1α or TFAM attenuates both mtROS induction and cisplatin induced apoptosis. Increasing mitochondrial ROS by inhibition or knock-down of the ROS-protective uncoupling protein UCP2 enhances cisplatin induced apoptosis. Similarly, enhancing ROS by high-dose ascorbic acid or H_2_O_2_ augments cisplatin induced apoptosis. In summary, mitochondrial content and the resulting mitochondrial capacity to produce ROS critically determine HGSC cell sensitivity to cisplatin induced apoptosis. In line with this observation, data from the human protein atlas (www.proteinatlas.org) indicates that high expression of mitochondrial marker proteins (TFAM and TIMM23) is a favorable prognostic factor in ovarian cancer patients. Thus, we propose mitochondrial content as a biomarker for the response to platinum-based therapies. Functionally, this might be exploited by increasing mitochondrial content or mitochondrial ROS production to enhance sensitivity to cisplatin based anti-cancer therapies.

## Introduction

Platinum compounds, i.e. cisplatin ([Pt(NH_3_)_2_Cl_2_]) or carboplatin ([Pt(C_6_H_6_O_4_)(NH_3_)_2_]) are among the most important agents for chemotherapeutic treatment of solid cancers. As such, platinum complexes are currently used as an inherent part of standard therapy for patients with high-grade serous ovarian cancer (HGSC)^1,2^. Commonly HGSC patients benefit from first-line therapy but eventually experience cancer recurrence. Hence, despite initial response ovarian cancer was, in the United States in 2017, the fifth leading cause of cancer death in women and the 5-year relative survival rate in patients with HGSC is as low as 46%^3^. In view of this evident treatment failure a deeper understanding of cellular response to platinum drugs is highly demanded to allow the identification of effective combinations to thwart resistance.

Altered energy metabolism of cancer cells compared to normal tissue was first shown by Otto Warburg^4^. The “Warburg effect” describes “aerobic glycolysis” as a biochemical phenomenon by which cancer cells generate energy from conversion of glucose to lactate even when properly oxygenated. Later it has become clear that not all tumor cells depend on “aerobic glycolysis” to generate energy as the oxygen supply is a function of the localization of the tumor cell within the tissue^5^. Intracellularly, energy in form of ATP is produced by mitochondria. These pivotal organelles also influence more distant cellular features ranging from stemness to differentiation and cell death (apoptosis)^6^. Apoptosis via the intrinsic signaling pathway crucially depends on mitochondrial release of cytochrome c, which acts as an electron transporter in energy metabolism. The context specific dual function of cytochrome c strikingly illustrates the intimate connection of energy metabolism and apoptosis. Consequently, “metabolic checkpoints” regulate induction of apoptosis and major cell death regulators like BAX and MCL-1 influence mitochondrial metabolism^7^.

Anticancer therapy by chemotherapeutic drugs commonly aims to compromise cellular integrity by damaging nuclear DNA thereby inducing cell death. Less prominent is the fact that mitochondrial DNA (mtDNA), like nuclear DNA (nDNA), is strongly affected by cisplatin. In fact cisplatin adducts of mitochondrial DNA are present at a 300- to 500-fold ratio as compared to nDNA^8^. Consequently, mtDNA-damage is evident in cisplatin treated cells^9^. The importance of mitochondria is readily evident when analyzing the response to cisplatin in cells with depleted mtDNA (ρ^0^ cells): Such ρ^0^ cells derived from the cisplatin-sensitive ovarian cancer cell line 2008 acquired resistance to cisplatin induced cell death^10^. In line with cell death critically depending on mtDNA-damage rather than nDNA-damage, apoptosis induction by cisplatin in testicular germ cell tumor cells does not require the nDNA-damage response mediating proteins ATM, ATR, or DNA-PK^11^. Even enucleated colon cancer cells still undergo apoptosis in response to cisplatin^12^.

Since several lines of evidence indicate that tumor sensitivity to cisplatin induced apoptosis is a function of mitochondria, we investigated the role of mitochondria in cisplatin induced apoptosis and elucidate the molecular basis for the involvement of mitochondria. Our analyses clearly show that cells respond to cisplatin by increasing the cellular amount of mitochondria, correlating directly with the sensitivity to cisplatin induced apoptosis. We propose that cisplatin mediated apoptosis driven by mitochondria is caused by cisplatin induced mitochondrial ROS that is detrimental to the cells and that the ROS level is a function of cellular mitochondrial content.

## Material and methods

### Cell culture and tumor samples

A2780, IGROV-1, OVCAR-3, OVCAR-4, OVCAR-5, and OVCAR-8 cells were purchased from the ATCC NCI-60 cell panel. All cells were cultivated in RPMI-1640 (Biochrom, Berlin, Germany) supplemented with 10% FCS and 10 mM l-glutamine. Cell cultures were routinely tested for mycoplasma.

### Reagents and experimental conditions

Cells were preincubated for 2 h with either 2 mM glutathione (Sigma-Aldrich, Munich, Germany), 50 µM zVAD-fmk (Bachem, Bubendorf, Switzerland), 5 µM Oligomycin A (BIOZOL, Eching, Germany), 2 mM ascorbic acid (Sigma-Aldrich), and 300 µM H_2_O_2_ (Otto Fischar GmbH, Saarbrücken, Germany). Then, cisplatin was added at 10 µM. Cells were simultaneously incubated with cisplatin and 100 µM Genipin (Sigma-Aldrich).

### Generation of monoclonal cell lines

500 cells were seeded into a coated 60 cm^2^ petri dish and cultured for 10–14 days. Evolving colonies were individually detached by trypsination using a silicon ring, seeded into 24-well plates and expanded.

### Detection of apoptosis

Annexin V-FITC (BD Pharmingen, Heidelberg, Germany) and propidium iodide (Sigma-Aldrich) staining was performed and measured by flow cytometry.

### Detection of mitochondrial content

Cells were stained with 200 nM Mitotracker Green (Thermo Fisher Scientific Inc.) or Acridine Orange 10-nonyl bromide (NAO) (Sigma-Aldrich) harvested and analyzed by flow cytometry.

### Oxygen consumption rate

After incubation in the presence or absence of above stated compounds cells were harvested by trypsination and 40.000 cells were seeded into a Seahorse Cell Culture Microplate, centrifuged and analyzed in a Seahorse XF96 Extracellular Flux Analyzer. A Seahorse XF Cell Mito Stress Test was performed with 1 µM Oligomycin A, 0.5 µM FCCP and 1 µM Antimycin A + Rotenone.

### Detection of mitochondrial ROS

Adherent cells were harvested by trypsination, counted and 0.1 × 10^6^ cells were stained with MitoSOX red (Thermo Fisher Scientific Inc.) and analyzed by flow cytometry.

### Measurement of cell division rate

Cells were seeded in a 6-well plate and harvested in 4 consecutive days for cell number determination.

### Detection of mitochondrial membrane potential

Cells were harvested, counted and 0.1 × 10^6^ cells were stained with TMRM (Thermo Fisher Scientific Inc.) and analyzed by flow cytometry.

### siRNA experiments

Gene expression of BAX, BAK, PGC1α, TFAM, or UCP2 was silenced using siGENOME SMARTpool siRNA (Horizon Discovery Ltd, Cambridge, GB). Control cells were transfected with siGenome Non-Targeting siRNA #1. In total 72 h after transfection using DharmaFECT#1 (Horizon Discovery Ltd), cells were incubated with 10 µM cisplatin 48 h. The efficacy of silencing was evaluated by western blot.

### Western blot analysis

Cells were harvested by trypsination, washed in ice cold PBS and lysed in 50 mmol/l Tris-HCl, 250 mmol/l NaCl, 0.1% Triton X-100, 5 mmol/l EDTA, pH 7.6, supplemented with cOmplete protease inhibitor cocktail (Roche, Grenzach-Wyhlen, Germany) and PhosSTOP phosphatase inhibitor cocktail (Roche) followed by sonication (50 cycles, 20 sec). Equal amounts of protein were diluted in 5xLaemmli buffer, heated (95 °C, 5 min.) and separated by SDS polyacrylamide gel electrophoresis. Proteins were transferred onto 0.45 µm nitrocellulose membrane. Incubation steps were: 1 h in blocking buffer (5% non-fat dry milk; 50 mmol/l Tris-HCl; 125 mM NaCl; pH 7.0) with shaking. Washing steps were 4 × 5 min in TBST; incubation with primary antibodies was over night at 4 °C, incubation with secondary antibodies was 1 h with shaking. Primary antibodies were as follows: anti-BAX #2772, anti-BAK #3814, anti-TFAM #8076, anti-UCP2 #89326, and anti-GAPDH (14C10) #2118 (Cell Signaling, Denvers, MA, USA); anti-PGC1α #ab54481 (Abcam, Cambridge, UK).

### TCGA data analysis

Survival data of the TCGA-OV project was downloaded from the TCGA Research Network: http://cancergenome.nih.gov/. Corresponding RNA expression data of the patients was obtained from the Human Protein Atlas Project (v15.0) (https://www.proteinatlas.org/ENSG00000108064-TFAM/pathology/tissue/ovarian+cancer) (https://www.proteinatlas.org/ENSG00000265354-TIMM23/pathology/tissue/ovarian+cancer)^13^.

### Statistical analysis

Data from at least three independent experiments are expressed as average ± standard deviation (SD). Statistical analysis was performed using GraphPad Prism 4.0 (GraphPad Software, La Jolla, USA). Changes in paired or unpaired samples were analyzed using two-sided paired or unpaired *t*-test and significance was considered when *p*-value < 0.05 (*p < 0.05; **p < 0.01; ***p < 0.001).

## Results

### Sensitivity to cisplatin induced cell death correlates with mitochondrial content

**Fig. 1:**
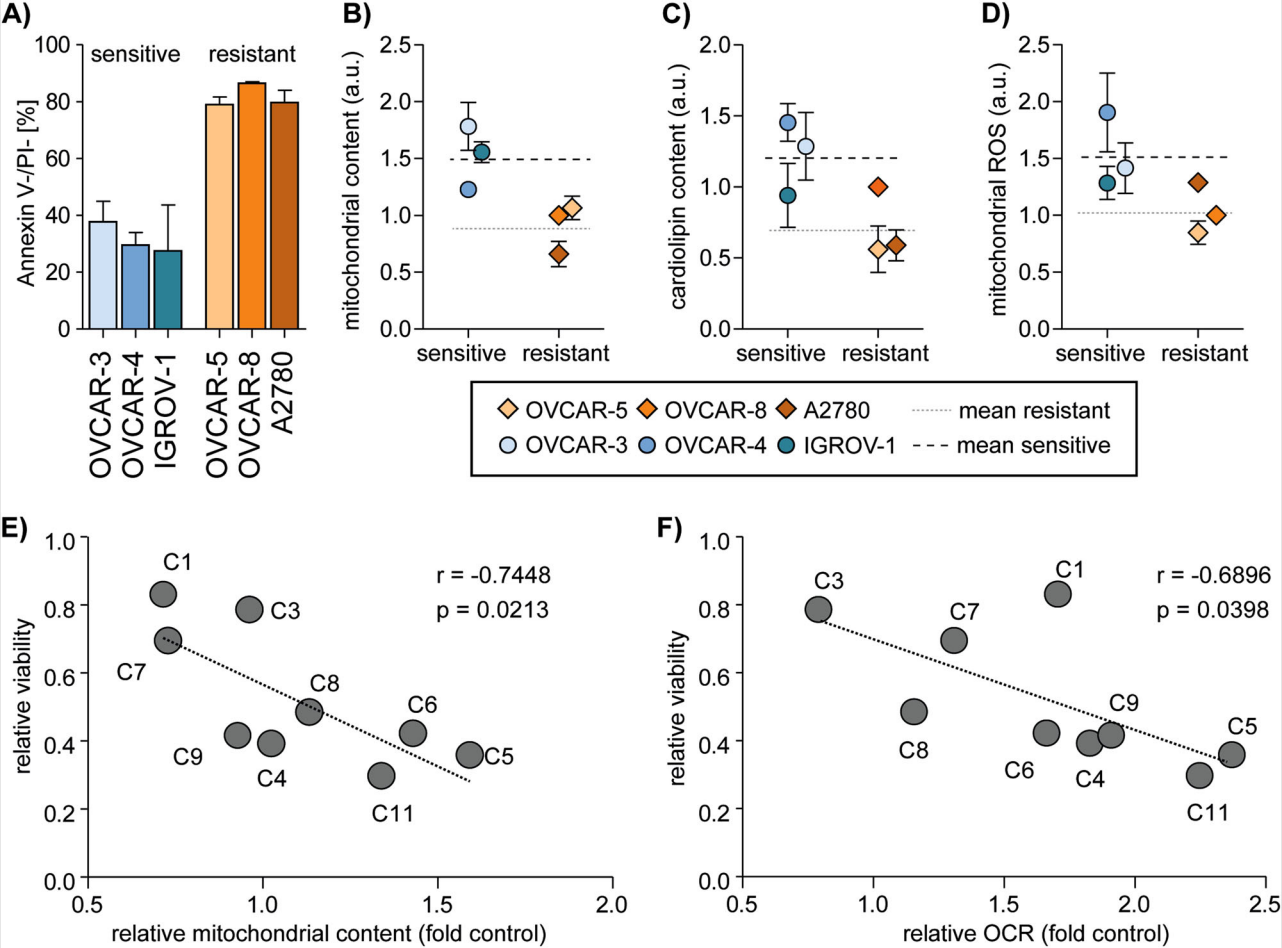
Mitochondrial content and mtROS correlate with cisplatin sensitivity. **a** Cell viability (Annexin V negative/PI negative) after 48 h treatment with 10 µM cisplatin reveals groups of apoptosis sensitive and resistant ovarian cancer cell lines. **b**–**d** Flow cytometric analysis show (**b**) increased mitochondrial content (Mitotracker Green), **c** increased cardiolipin content (Acridine Orange 10-nonyl bromide) and **d** increased mitochondrial ROS (MitoSOX red) in apoptosis sensitive cell lines. Data was normalized to resistant cell line OVCAR-8. **e**, **f** Single clones of OVCAR-3 were picked, expanded for 2 weeks and flow cytometrically analyzed for **e** mitochondrial content (Mitotracker Green) compared to the parental cell line, for viability (AnnexinV/PI negative) after 48 h of 10 µM cisplatin treatment normalized to untreated cells and **f** analyzed in a Seahorse XF96 Extracellular Flux Analyzer for basal oxygen consumption rate compared to the parental cell line. Correlation analysis was performed with GraphPad Prism 4.0. Data represent means ± SD from at least three independent experiments

Platinum based chemotherapeutic compounds critically target nuclear DNA integrity. Less well studied is the impact of cisplatin on mitochondria. To clarify the role of mitochondrial functionality in cisplatin-resistance of ovarian cancer cells we first analyzed cellular sensitivity to cisplatin induced cell death in high-grade ovarian cancer cell lines from the NCI-60 panel. Cells from six cell lines (OVCAR-3, OVCAR-4, OVCAR-5, OVCAR-8, IGROV-1, and A2780) were incubated in the presence of 10 µM cisplatin for 48 h and induction of apoptosis was analyzed by Annexin V-FITC/PI staining and flow cytometric analysis (Fig. 1a). Cisplatin efficiently induced cell death in OVCAR-3, OVCAR-4, and IRGOV-1 (>60% cell death) whereas OVCAR-5, OVCAR-8, and A2780 showed <30% apoptotic cells in response to cisplatin. Based on these results we grouped the cells in “sensitive” (OVCAR-3, OVCAR-4, and IGROV-1) and “resistant” (OVCAR-5, OVCAR-8, and A2780). To further analyze the role of mitochondria in cisplatin induced cell death we assessed the relative mitochondrial content by staining the cells with Mitotracker Green and subsequent flow cytometric analysis (Fig. 1b). These analyses show that the sensitive cell lines are characterized by higher relative mitochondrial content (mean 1.52) than the resistant cell lines (mean 0.91). To confirm the disparate mitochondrial content of sensitive and resistant cells we detected the mitochondria specific lipid cardiolipin using 10-N-nonyl acridine orange (NAO) in flow cytometric analysis (Fig. 1c). Again, a higher relative content of cardiolipin indicating higher mitochondrial content was observed in sensitive cell lines as compared to resistant cell lines. Hence, higher mitochondrial content directly correlates with sensitivity to cisplatin induced apoptosis. As mitochondrial reactive oxygen species are known to be toxic, we next analyzed the cellular levels of mitochondrial reactive oxygen species (mtROS) by MitoSOX red staining (Fig. 1d). Expectedly, the sensitive ovarian carcinoma cell lines were characterized by higher mtROS as compared to the resistant cell lines. We also analyzed in a Seahorse XF96 Extracellular Flux Analyzer the mitochondrial basal oxygen consumption rate (OCR). A higher OCR was detected in the sensitive than in the resistant cell lines (Fig. S1a), which is in concordance with a higher mitochondrial content.

In order to further analyze the impact of mitochondrial content in a genetically constant background we generated clonal sublines from the cisplatin sensitive OVCAR-3 cell line. Despite genetic identity, these subclones show disparate mitochondrial content and disparate sensitivity to cisplatin induced cell death^14^. In agreement with our previous results, these OVCAR-3 subclones display a significant correlation of the relative mitochondrial content and their sensitivity to cisplatin induced apoptosis (Fig. 1e). Similar to the relative mitochondrial content also the OCR correlated with sensitivity to cisplatin induced apoptosis (Fig. 1f). These data show a clear correlation of mitochondrial activity and cellular sensitivity to cisplatin induced apoptosis not only in different cell lines but also in genetically similar sublines generated from a common precursor cell line.

### Cisplatin induced cell death depends on ROS, BAX&BAK and caspases but is independent of proliferation of the cells

**Fig. 2:**
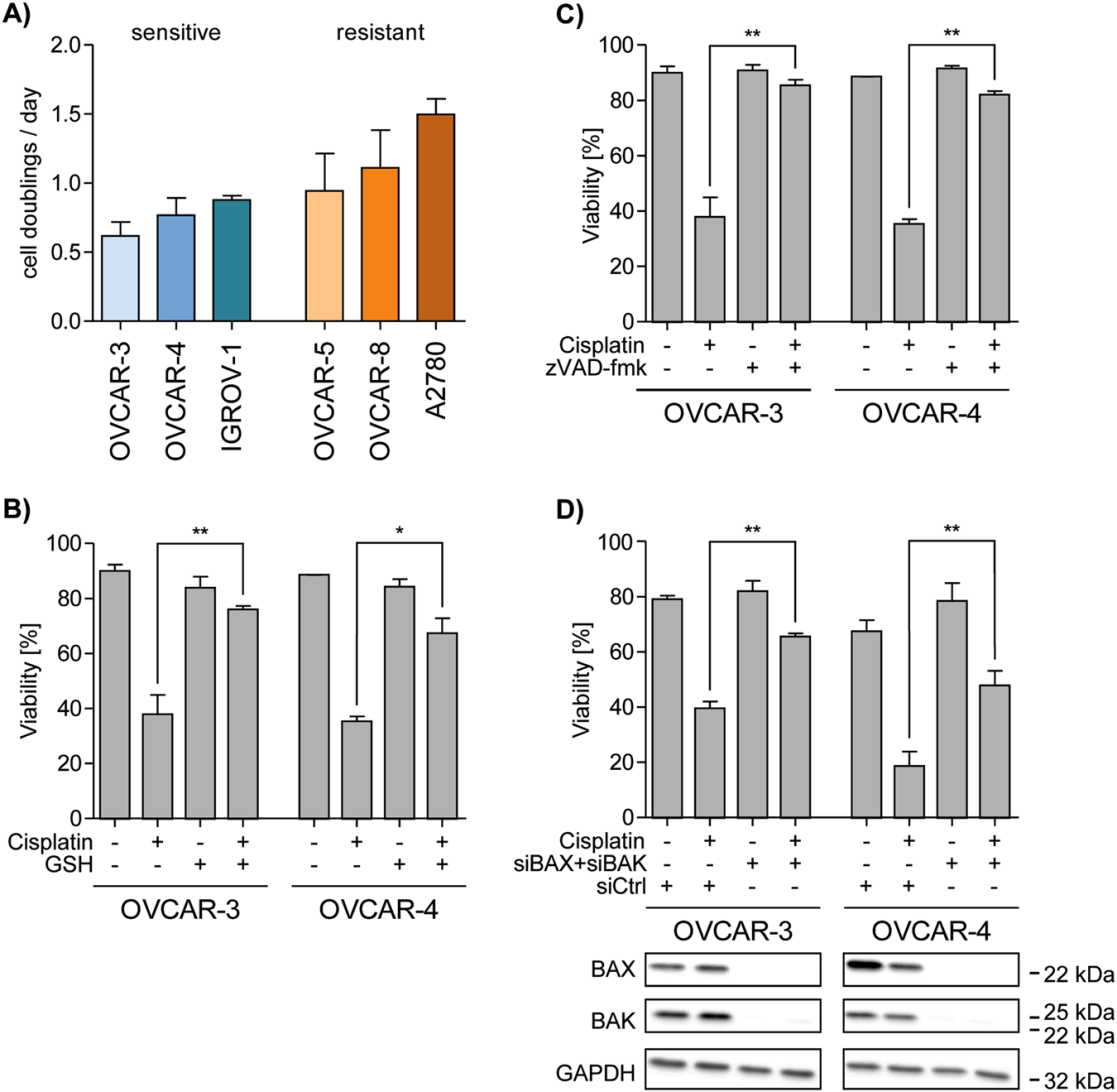
Cisplatin induces intrinsic and ROS dependent apoptosis. **a** Division rate was determined by counting cells on 4 consecutive days. **b**, **c** Viable (Annexin V negative/PI negative) cells after co-incubation with **b** 2 mM Glutathion or **c** 50 µM zVAD-fmk and 10 µM cisplatin for 48 h. (**d**, bottom) Western blot analysis of BAX and BAK expression in cells transfected with siRNA targeting BAX and BAK or control siRNA and (**d**, top) flow cytometric analysis of viability (Annexin V negative/PI negative) after treatment with 10 µM cisplatin for 48 h. Data represent means ± SD from at least three independent experiments

Nuclear DNA-damage induction by chemotherapeutic drugs is particularly toxic for rapidly proliferating cells. Intriguingly, we found that the cisplatin sensitive cell lines OVCAR-3, OVCAR-4, and IGROV-1 show a slightly lower proliferation rate than the resistant cell lines OVCAR-5, OVCAR-8, and A2780 (Fig. 2a). Thus, in addition to nuclear DNA-damage induction by cisplatin its known effect to induce production of ROS in target cells might impact on cell death induction^15^. To analyze the relevance of ROS in cisplatin induced cell death we compared viability of cells that had been incubated with cisplatin in the presence or absence of the ROS scavenger glutathione (GSH) (Fig. 2b). Incubation with GSH did not affect the viability of OVCAR-3 and OVCAR-4 cells but efficiently enhanced viability of cells incubated with cisplatin: OVCAR-3 (38% vs 75%); OVCAR-4 (35% vs 67%). In an analogous experiment we verified that cisplatin induced cell death is apoptotic rather than necrotic by incubating cells with the pan-caspase inhibitor zVAD-fmk (Fig. 2c). Inhibition of caspase-activity completely blocked cell death induction verifying apoptotic cell death. Acceptedly, apoptosis mediated by the mitochondrial signaling pathway critically depends on the pore-forming effector proteins BAX and/or BAK^16^. To provide further proof for mitochondria-mediated apoptosis we employed small-interfering RNA to knock-down the effector proteins BAX&BAK and subsequently analyzed cisplatin induced apoptosis. In line with cell death induction via the mitochondrial apoptosis pathway, knock-down of BAX&BAK (Fig. 2d, lower panel) efficiently rescued cells from cisplatin induced cell death (Fig. 2d, upper panel). These data show that cell death induction by cisplatin depends on the induction of ROS, the presence of pore-forming BAX&BAK and active caspases.

### Cisplatin induces mtROS, enhances mitochondrial content and increases mitochondrial membrane potential ΔΨm

**Fig. 3:**
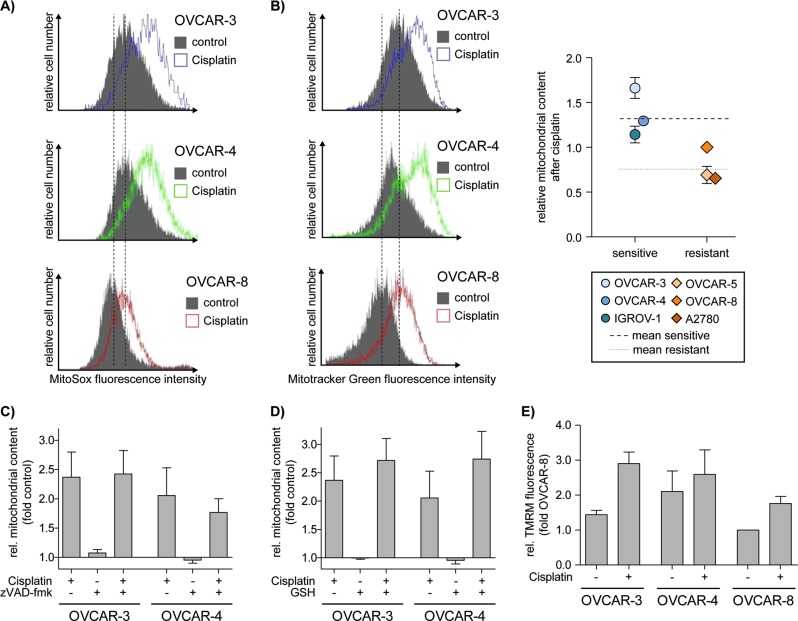
Cisplatin induces mtROS and enhances mitochondrial content. **a**, **b** Flow cytometric analysis of OVCAR-3, OVCAR-4 and OVCAR-8 show increased (**a**) mtROS (MitoSOX red) and (**b**) mitochondrial content (Mitotracker Green) after treatment with 10 µM cisplatin for 48 h. The cisplatin sensitive cell lines have more mtROS and mitochondrial content (**b**, right) than the cisplatin resistant cell lines before and after incubation with cisplatin (means indicated by dotted lines). **c, d** Flow cytometric analysis shows that induction of mitochondrial content (Mitotracker Green) after cisplatin treatment is not affected by (**c**) 50 µM zVAD-fmk or (**d**) 2 mM Glutathione in OVCAR-3 and OVCAR-4 cells. **e** Flow cytometric analysis reveals higher mitochondrial membrane potential (TMRM) in cisplatin sensitive cell lines OVCAR-3 and OVCAR-4 than in resistant OVCAR-8. Cells treated with 10 µM Cisplatin for 48 h have increased mitochondrial membrane potential. Data represent means ± SD or a representative experiment from at least three independent experiments

Cisplatin is a well-known inducer of ROS^17–19^ but the intracellular origin of ROS production is not fully understood. An important source of ROS in mammalian cells are mitochondria^20^ and especially cisplatin induced ROS was shown to be produced by mitochondria^21^. We analyzed cisplatin induced ROS production in the sensitive cell lines OVCAR-3 and OVCAR-4 and the resistant cell line OVCAR-8 (Fig. 3a). Induction of ROS by cisplatin was detected in all cell lines, however, the sensitive OVCAR-3 and OVCAR-4 cells showed higher ROS production compared to the resistant OVCAR-8 cells under control conditions as well as in response to cisplatin (vertical lines). Similarly, increased ROS was detected by CellROX Green or CellROX Deep Red, which have slightly altered specificity for cellular ROS as compared to MitoSOX (Fig. S1b, c). Because the sensitive cells also showed higher mitochondrial content (Fig. 1b, c) we wondered whether cisplatin induced ROS production also correlated with a further increase in mitochondrial content. Indeed, analysis of mitochondrial content by staining of the cells with Mitotracker Green clearly indicated an increase of mitochondrial content in response to cisplatin in sensitive OVCAR-3 and OVCAR-4 but also in resistant OVCAR-8 cells (Fig. 3b, left panel). Noteworthy, the peak mitochondrial content was consistently higher in sensitive cells as compared to cells resistant to cisplatin induced apoptosis (Fig. 3b, right panel). In order to delineate whether increased mitochondrial content is cause or consequence of cisplatin induced ROS production or apoptosis we again incubated cells with the ROS scavenger GSH or the pan-caspase-inhibitor zVAD-fmk. Subsequent flow cytometric analysis showed that neither GSH nor caspase-inhibition prevents the cisplatin induced increase in mitochondrial content. Hence, we conclude that cisplatin induces an increase of mitochondrial content upstream of ROS production and caspase activity (Fig. 3c, d). In line with upstream induction of mitochondrial content, TMRM staining showed increased overall mitochondrial membrane potential (ΔΨm) in OVCAR-3, OVCAR-4, and OVCAR-8 cells (Fig. 3e). In line with cisplatin induced increase of mitochondrial content, cisplatin also increased the basal OCR and the maximal respiration in OVCAR-3 and OVCAR-8 cells (Fig. S1d). Taken together, cisplatin treatment of ovarian cancer cell lines induces increased mitochondrial content, enhanced OCR and mitochondrial membrane potential and higher levels of mitochondrial ROS. Noteworthy, induction of mitochondrial content is upstream of ROS induction and caspase activation.

### Mitochondrial content and ROS are causally linked to cisplatin induced cell death

**Fig. 4:**
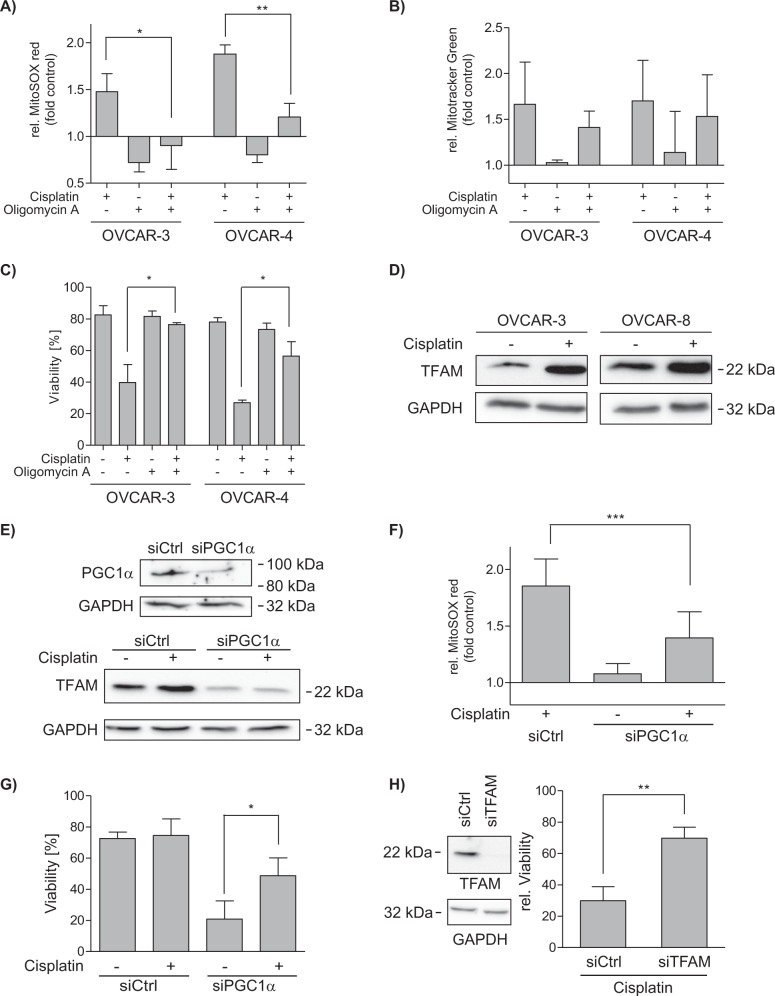
Reduction of mtROS enhances survival after cisplatin treatment. **a**–**c** Flow cytometric analysis of OVCAR-3 and OVCAR-4 after co-treatment with ATP synthase inhibitor Oligomycin A (5 µM) and 10 µM cisplatin for 48 h. **a** Oligomycin A reduces cisplatin mediated mtROS induction (MitoSOX red) while **b** mitochondrial content (Mitotracker Green) is unaffected. **c** Oligomycin A increases viability (Annexin V negative/PI negative) after cisplatin incubation. **d** Western blot analysis of OVCAR-3 and OVCAR-8 cells after treatment with 10 µM cisplatin for 48 h shows increased TFAM protein expression. **e**–**g** siRNA mediated knockdown of PGC1α in OVCAR-4. **e** Expression of PGC1α (top) and TFAM (bottom) are decreased in transfected OVCAR-4 cells. **f** Flow cytometric analysis shows decreased mtROS induction (MitoSox red) in PGC1α silenced cells after treatment with 10 µM cisplatin for 48 h. **g** Flow cytometric analysis of cell viability (Annexin V negative/PI negative) reveals PGCA1 α knockdown mediated enhanced survival after treatment with 10 µM cisplatin for 48 h. (**h**, left) siRNA mediated knockdown of TFAM in OVCAR-4 cells (**h**, right) is associated with enhanced viability (Annexin V negative/PI negative) in a flow cytometric analysis after treatment with 10 µM cisplatin for 48 h. Data represent means ± SD or a representative experiment from at least three independent experiments

To further elucidate the role of elevated mitochondrial activity in response to cisplatin treatment, we blocked mitochondrial function by the ATP synthase inhibitor Oligomycin A. After 48 h, the OCR was reduced and could not be reduced any further by additional injection of Oligomycin A in the “MitoStress Test” (Supplementary Fig. S2a). Inhibition of ATP synthase during cisplatin treatment prevented the induction of mtROS (Fig. 4a) and also increase of the mitochondrial membrane potential (Supplementary Fig. S2b), whereas mitochondrial content was still amplified (Fig. 4b). Diminished ROS induction was accompanied by significant reduction of cisplatin induced apoptosis (Fig. 4c), showing the importance of increased mtROS for apoptosis induction.

The mitochondrial transcription factor TFAM drives the mitochondrial transcription machinery^22^ and regulates mitochondrial biogenesis^23,24^. Expression of TFAM was increased in sensitive OVCAR-3 and resistant OVCAR-8 cells after 48 h incubation with cisplatin which is in line with mitogenesis (Fig. 4d). Mitogenesis associated expression of TFAM is regulated by PGC1α (Peroxisome proliferator-activated receptor gamma coactivator 1-alpha), the master regulator of mitochondrial biogenesis^25^. A partial knock-down of PGC1α prevented the cisplatin induced increase of TFAM protein expression (Fig. 4e). Moreover, knock-down of PGC1α reduced ROS induction after cisplatin treatment (Fig. 4f) and significantly protected sensitive OVCAR-4 cells from cisplatin induced apoptosis (Fig. 4g). In line, also knock-down of TFAM prevented cisplatin induced apoptosis (Fig. 4h). Overall, blocking mitochondrial function or preventing mitochondrial biogenesis causes a decrease of mtROS induction by which sensitive ovarian cancer cells can be rescued from cisplatin induced cell death.

### Mitochondrial content is a prognostic factor for survival of ovarian cancer patients

**Fig. 5:**
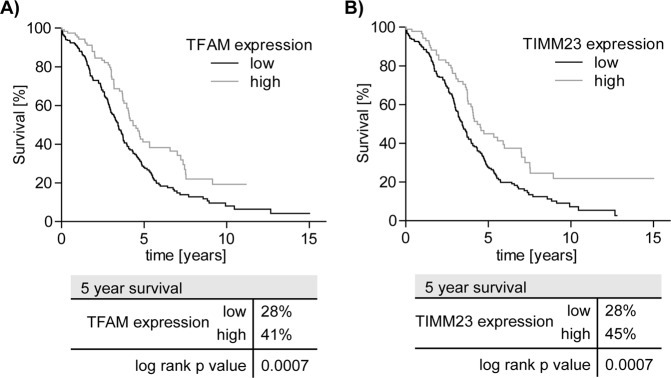
Mitochondrial content correlates with better 5-year survival of OVCAR patients. **a**, **b** Survival data analysis of the TCGA-OV project. **a** Kaplan–Meier plot of patients with high or low TFAM expression with cut off at 10.82 FPKM. **b** Kaplan–Meier plot of patients with high or low TIMM23 expression with cut off at 61.81 FPKM. Data obtained from the TCGA Research Network (http://cancergenome.nih.gov) and the human protein atlas (www.proteinatlas.org)

The presented data shows that a key element of cisplatin induced cell death is mitochondrial ROS and mitochondrial content. The expression of mitochondrial transcription factor TFAM and Translocase Of Inner Mitochondrial Membrane 23 (TIMM23) reflect mitochondrial content and might therefore be indicative for response of HGSC to cisplatin based therapy, which still is the first-line therapy of ovarian cancer after surgery^1,2^. Hence, it is conceivable that TFAM and TIMM23 expression – as surrogate markers for mitochondrial content – correlate with enhanced overall survival. We investigated TFAM and TIMM23 expression and overall survival in the TCGA-OV (v15.0) and the project data indeed shows significantly (*p* < 0.005) higher 5-year survival probability for patients with high expression of TFAM (41%) or TIMM23 (45%) as compared to those with low TFAM (28%) or TIMM23 (28%) expression (Fig. 5a, b). Thus, we propose that high expression of mitochondrial proteins like TFAM and TIMM23 are favorable prognostic factors for HGSC patients because their expression correlates with mitochondrial content and mtROS production, which in turn amplifies sensitivity to cisplatin induced apoptosis. Intriguingly, highest expression of TFAM and TIMM23, among all tissues, is found in testis (www.proteinatlas.org;^13^) – and testis cancer is commonly cured by cisplatin therapy.

### Increase of mtROS overcomes cisplatin resistance

**Fig. 6:**
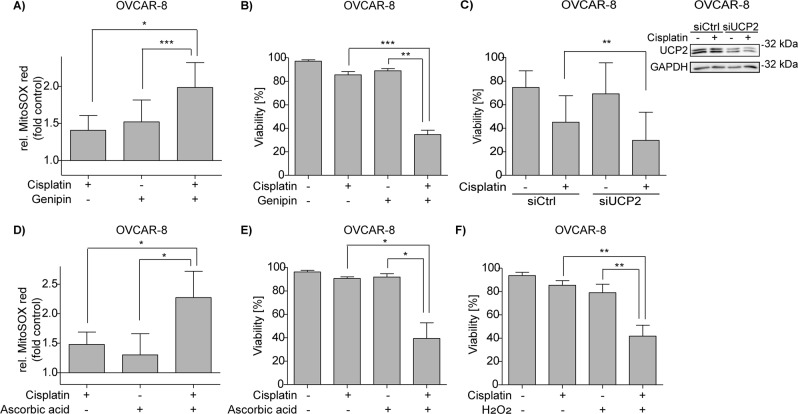
Increase of mtROS in combination with cisplatin enhances cell death in resistant cells. **a**, **b** Flow cytometric analysis of the cisplatin resistant cell line OVCAR-8 after co-treatment with 100 µM Genipin and 10 µM cisplatin for 48 h. **a** Genipin increases cisplatin mediated mtROS induction (MitoSOX red). **b** Viability (Annexin V negative/PI negative) of OVCAR-8 cells is reduced by co-incubation with Genipin and cisplatin. **c** Knock-down of UCP2 sensitizes OVCAR-8 to cisplatin induced apoptosis. **d**, **e** Flow cytometric analysis of OVCAR-8 cells after co-treatment with 2 mM ascorbic acid and 10 µM cisplatin for 48 h. **d** Ascorbic acid increases cisplatin mediated mtROS induction (MitoSOX red). **e** Viability (Annexin V negative/PI negative) of OVCAR-8 cells is reduced by co-incubation with ascorbic acid and cisplatin. **f** Flow cytometric analysis reveals reduced viability (Annexin V negative/PI negative) of OVCAR-8 cells after co-treatment with 300 µM H_2_O_2_ and 10 µM cisplatin for 48 h. Data represent means ± SD from at least three independent experiments

We show that inhibition of mitochondrial function and mitogenesis enhance the survival of cisplatin sensitive cells and that sensitivity to cisplatin induced apoptosis correlates with mitochondrial content and mtROS. Increased mtROS induces activation of counteracting “uncoupling proteins” (UCP) that decrease mtROS production and mitochondrial membrane potential by mediating leakage of protons through the mitochondrial inner membrane^26–28^. We analyzed proton leakage using the MitoStress Test and found increased proton leakage in response to cisplatin (Supplementary Fig. S3) in OVCAR-8 cells. Hence, we thought to exploit these mechanistic insights to improve anti-cancer therapy and asked whether Genipin, an inhibitor of UCP2, enhances cellular sensitivity to cisplatin induced apoptosis. In accordance with the above outlined mechanisms, Genipin significantly increased cisplatin induced mitochondrial ROS in the resistant cell line OVCAR-8 (Fig. 6a) and also enhanced cell death (Fig. 6b). In an alternative approach we aimed to increase cisplatin induced apoptosis by knocking-down the ROS protective UCP2. Also, reduced UCP2 expression clearly resulted in enhanced sensitivity to cisplatin induced apoptosis (Fig. 6c). Increased intracellular ROS acceptedly is also induced by high concentration of ascorbic acid^29,30^. At 2 mM concentration ascorbic acid caused a minor increase of mtROS levels in OVCAR-8 while ascorbic acid together with cisplatin strongly enhanced mtROS levels (Fig. 6d) resulting in cell death of the resistant OVCAR-8 cells (Fig. 6e). In order to verify that ROS are causally linked to cisplatin induced cell death OVCAR-8 cells were incubated with cisplatin in combination with H_2_O_2_. Although exogenous H_2_O_2_ (300 µM) did not significantly affect cell viability, exogenous ROS clearly aided in cisplatin induced cell death (Fig. 6f). Increasing ROS by pharmacological means might potentially sensitize ovarian cancer cells to cisplatin and overcome resistance.

## Discussion

Platinum compounds have been used in anti-cancer therapy for decades and their damaging activity on nDNA is well characterized. However, focusing on the nuclear DNA-damage response does not show the entire effect of cisplatin on cells given that there is an additional pool of DNA in cellular organelles, i.e. mitochondria. Undoubtedly, mitochondria are a central component of intrinsic apoptosis signaling. Not surprisingly mitochondria play an important role in chemo-resistance^31–33^. Interestingly, cisplatin induced damage of mtDNA^8,10^ affects mitochondrial function and enhances cell death^21^. Therefore, we aimed to investigate the effect of cisplatin on mitochondria and the role of mitochondria in the cellular response to cisplatin in ovarian cancer cells. For the first time we present evidence that cisplatin sensitive ovarian cancer cell lines (OVCAR-3, OVCAR-4, and IGROV-1) show higher mitochondrial content and more mtROS than less sensitive or resistant cell lines (OVCAR-5, OVCAR-8, and A2780). Furthermore, clonal and hence most likely isogenic sublines of cisplatin sensitive OVCAR-3 cells confirmed these results, because even in these sublines the relative mitochondrial content and OCR correlate with susceptibility to cisplatin induced cell death. These results are in line with observations that cisplatin resistant ovarian cancer cell line C13 present with reduced mitochondrial function and enhanced dependency on glycolysis and the pentose phosphate pathway than their sensitive counterpart 2008^10,33^. Consequently, depletion of mitochondrial DNA in ρ^0^ cells mediates resistance to cisplatin induced cell death^8,10^.

Cisplatin induces intrinsic apoptosis that depends on pore-forming pro-apoptotic Bcl-2 family proteins BAX&BAK and caspase activation. More importantly, we find that cisplatin mediated activation of the intrinsic apoptosis pathway depends on mitochondrial ROS. In line, cisplatin induced apoptosis even independent of nuclear DNA is illustrated by the fact that testicular germ cell tumor cells still undergo cisplatin induced apoptosis in the absence of key mediators of DNA damage response, such as ATM, ATR, or DNA-PK^11^. Furthermore, enucleated colon cancer cells undergo apoptosis in response to cisplatin^12^, thus placing mitochondria into the focus of cisplatin induced apoptosis.

Mechanistically, mitochondria likely play a dual role in cisplatin induced apoptosis: while BAX&BAK mediated permeabilization and release of cytochrome c is indispensable for the induction of the effector phase of apoptosis, we show a critical function of cisplatin induced increase of mitochondrial content and mtROS^21^ upstream of BAK&BAK oligomerization. We present evidence by various methods that cisplatin enhances cellular mitochondrial content (e.g. Fig. 3b, S1d). However, a remaining question still is whether (a) a subpopulation of damaged mitochondria produces increased mtROS or (b) the higher amount of mtROS originates from basal mtROS production by increased mitochondrial content. We here show that increased mitochondrial content correlates with increased mtROS and enhanced sensitivity to cisplatin induced cell death. We propose that mitochondrial content and cisplatin induced mitogenesis influence sensitivity to cisplatin induced apoptosis. We confirm this by showing that (a) blocking ATP synthase with Oligomycin A blocks mtROS production and (b) blocking mitochondrial biogenesis by knock-down of PGC1α or TFAM reduces cisplatin induced cell death. Reduced mitochondrial content alleviates mtROS and renders sensitive cells resistant to cisplatin induced cell death. On the other hand, increase of mitochondrial ROS by Genipin mediated blocking of UCP2 or knock-down of UCP2, treatment with high doses of ascorbic acid or the application of H_2_O_2_ enhance cisplatin induced cell death. The role of mtROS in cisplatin induced apoptosis is illustrated by reduced toxicity of carboplatin that does induce far less mtROS^21^. Production of mtROS can be enhanced by tuning mitochondrial metabolism, e.g. by dichlor acetate that shift mitochondria from glycolysis to glucose oxidation^34^ resulting in enhanced sensitivity to cisplatin^21^. Likewise, cultivation of cells in glucose-free medium complemented with galactose increases mitochondrial activity^35^. Therefore, cultivation in galactose-supplemented medium, not surprisingly, renders resistant ovarian cancer cells sensitive to cisplatin induced cell death^36^.

**Fig. 7:**
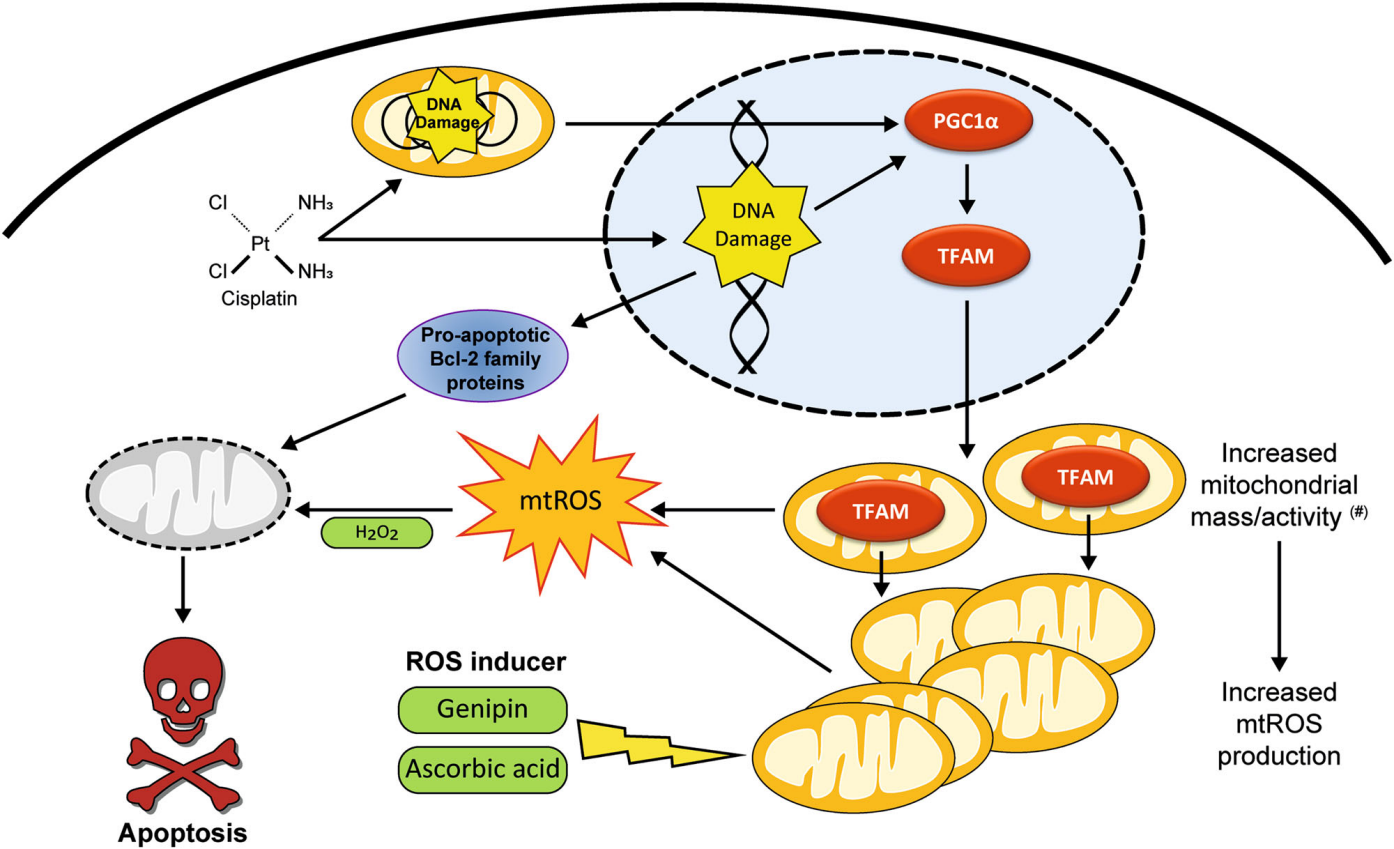
Model of cisplatin-induced cell death in ovarian cancer cells. Cisplatin causes nDNA and mtDNA damage. The latter results in the activation of PGC-1α and increased expression of TFAM. TFAM translocates to mitochondria and causes mitochondrial biogenesis and activity, which in turn leads to increased mtROS production causing cell death in sensitive cell lines. By further increasing mtROS with Genipin, ascorbic acid or H_2_O_2_ resistant cells are sensitized to cisplatin induced cell death. (#) Noteworthy, the depicted model for cisplatin-induced mtROS is non-exclusive and certainly other mechanisms, such as impairment of ETC function^21^, are involved in the production of mtROs

Cisplatin treatment impairs the integrity mtDNA^8,37^, induces mtROS and thereby causes reduced energy production by generation of dysfunctional mitochondria^15^. Mitochondrial dysfunction and mtROS in turn enhance mitochondrial biogenesis by inducing the expression of PGC1α, nuclear respiratory factors 1 and 2 (Nrf1, Nrf2), and TFAM^38^. Since intact mitochondria during respiration partially convert consumed oxygen to mtROS^39^ it is obvious that the cisplatin induced increased mitochondrial content inevitably results in higher levels of mtROS. However, previous studies show that cisplatin induced mtROS results from inhibition of transcription of mtDNA-encoded proteins, resulting in impairment of ETC function^21^ (Fig. 7 (#)). In any case, because the level of mtROS positively influences sensitivity to cisplatin induced apoptosis induction it is worthwhile to investigate whether pharmacological compounds that increase mitochondrial biogenesis and/or mtROS or decrease the activity of the anti-oxidative machinery are suitable to enhance the efficacy of cisplatin based anti-cancer therapy or even overcome resistance in ovarian cancer patients (Fig. 7). This aim might be achieved by modern pharmacological intervention using genipin (Fig. 6a, b) or more unconventional approaches such as high-dose ascorbic acid (Fig. 6d, e).

Undoubtedly, the relevance of mitochondrial content for therapy response is evidenced by RNA expression analyses available in TCGA-OV data that show superior overall survival of ovarian cancer patients with high expression of the mitochondrial proteins TFAM and TIMM23. Also, in line with the proposed role of mitochondrial content and mtROS production in response to cisplatin based anticancer therapy, analysis of the TCGA-OV data indicates a favorable therapy response of patients with low expression of UCP2^40^, a protein that reduces production of mtROS. In summary, we provide solid evidence for a highly relevant function of mitochondria in cisplatin based anti-cancer therapy beyond and yet intricately entangled with their indispensable role in the intrinsic apoptosis signaling pathway. The mechanistic insights presented here establish mitochondria as new marker structures for the prediction of therapy response. Furthermore, improved therapy response might be achievable by combining cisplatin based drugs with substances that impair efficient function of ROS-protective mechanisms, either by pharmacological or physiological means such as hyper-oxygenation.

## Supplementary information

Supplementary Figure Legends

Supplementary Figure S1

Supplementary Figure S2

Supplementary Figure S3
