# Supplementary material for: Direct impact of cisplatin on mitochondria induces ROS production that dictates cell fate of ovarian cancer cells
Source: Cell Death Dis. 2019 Nov 7;10(11):851. doi: 10.1038/s41419-019-2081-4 (PMC6838053; doi:10.1038/s41419-019-2081-4)
Supplement: Supplementary file 1 — Supplementary Figure Legends [file 41419_2019_2081_MOESM1_ESM.docx]

**Figures S1: OCR is higher in cell lines that are sensitive to cisplatin induced cell death and cisplatin induces cellular ROS. a, d** Analysis of OCR in a Seahorse XF96 Extracellular Flux Analyzer reveals that (**a**) basal OCR is higher in sensitive cell lines as compared to the resistant cell lines. Flow cytometric analysis of OVCAR-3, OVCAR-4 and OVCAR-8 show enhanced cellular ROS with (**b**) CellROX Green and (**c**) CellROX Deep Red staining after incubation with 10 µM cisplatin for 48 h. Higher cellular ROS is detected in sensitive cell lines OVCAR-3 and OVCAR-4 than in cisplatin resistant cell line OVCAR-8 before and after incubation with cisplatin (means indicated by dotted lines). **d** Basal OCR and maximal respiration (Maximal respiration = OCR after FCCP injection minus OCR after Antimycin A/Rotenone injection) is increased in OVCAR-3 and OVCAR-8 cells after incubation with 10 µM cisplatin for 48 h. Data represent means ± SD or a representative experiment from at least three independent experiments.

**Figures S2: ATP synthase inhibition by Oligomycin A prevents cisplatin mediated increase of mitochondrial membrane potential. a** Analysis of OCR in a Seahorse XF96 Extracellular Flux Analyzer of OVCAR-3 cells after co-treatment with 5 µM Oligomycin A and 10 µM cisplatin for 48 h. 5 µM Oligomycin A completely blocks ATP synthase. **b** Flow cytometric analysis of OVCAR-3 and OVCAR-4 cells after co-incubation with 5 µM Oligomycin A and 10 µM cisplatin for 48 h shows significant reduction of the mitochondrial membrane potential (TMRM). Data represent means ± SD or a representative experiment from at least three independent experiments.

**Figures S3: Cisplatin increases proton leak.** Analysis of OCR in a Seahorse XF96 Extracellular Flux Analyzer of OVCAR-8 cells after treatment with 10 µM cisplatin for 48 h shows increased proton leak (Proton leak = OCR after inhibition of the ATP synthase with Oligomycin A minus non-mitochondrial respiration).
