# Supplementary figures and images for: Direct impact of cisplatin on mitochondria induces ROS production that dictates cell fate of ovarian cancer cells
Source: Cell Death Dis. 2019 Nov 7;10(11):851. doi: 10.1038/s41419-019-2081-4 (PMC6838053; doi:10.1038/s41419-019-2081-4)

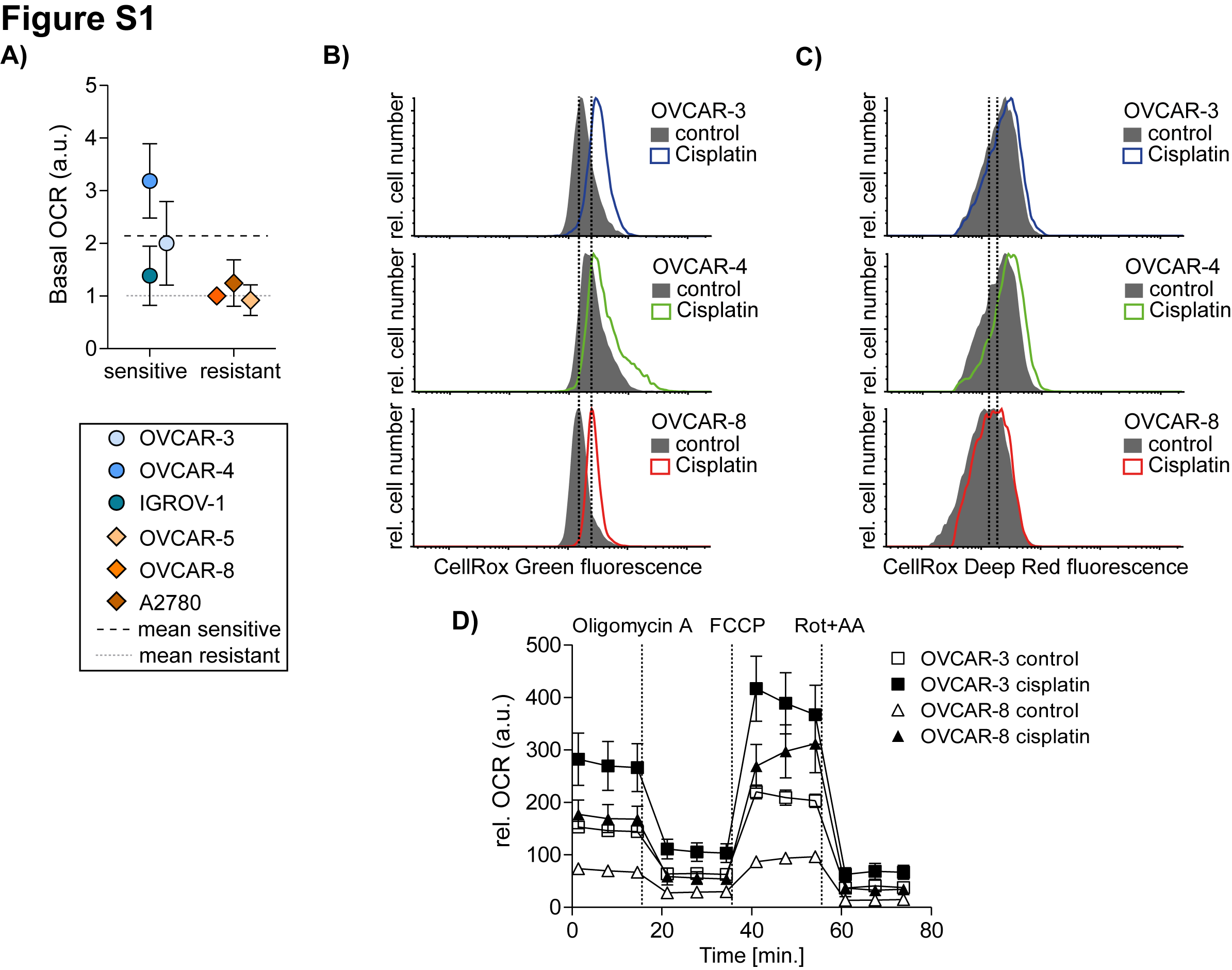

Supplement: Supplementary file 2 — Supplementary Figure S1 [file 41419_2019_2081_MOESM2_ESM.tif]

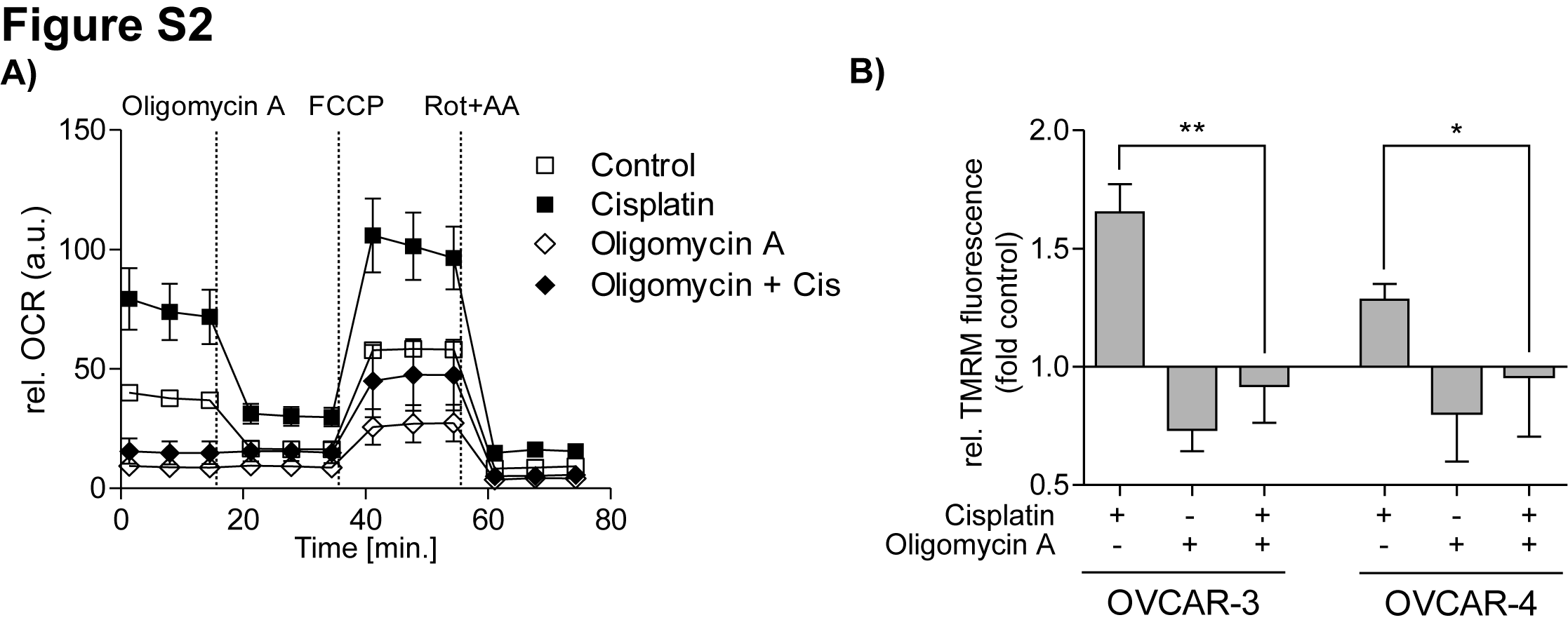

Supplement: Supplementary file 3 — Supplementary Figure S2 [file 41419_2019_2081_MOESM3_ESM.tif]

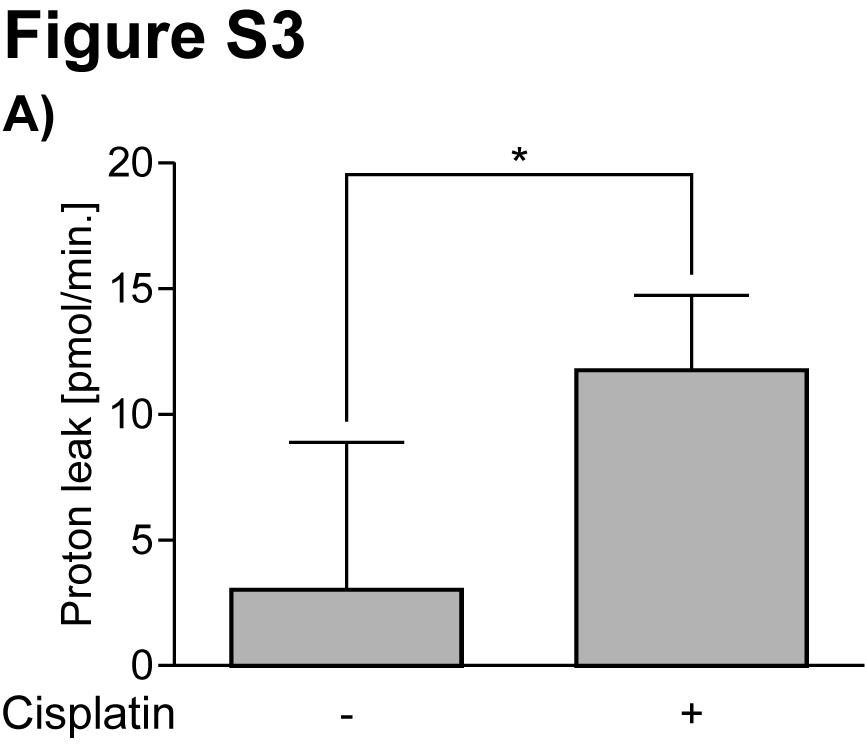

Supplement: Supplementary file 4 — Supplementary Figure S3 [file 41419_2019_2081_MOESM4_ESM.tif]
